# Errors in Dr. T. F. Allen’s Encyclopedia and Index

**Published:** 1890-09

**Authors:** E. W. Berridge

**Affiliations:** London, England


### ERRORS IN DR. T. F. ALLEN'S ENCYCLOPÆDIA AND INDEX.

E. W. Berridge, M. D., London, England.

As this work is at present our only completed (?) *Materia Medica and Index*, physicians ought to be informed of some of the errors therein.

In *Iodine*, proving 14, by Dr. C. Mohr, really belongs to *Indium*.

In *Antim-tart.*, proving 1, from Hartlaub and Trinks, is said by Dr. Dudgeon to belong to *Manganum*, but I have not the original to refer to.

In the *Repertory* I have frequently found the following confused: K-n. (*Kali-nitricum*), with Kre. (*Kreosote*); Alum (*Alumina*), with Alumn. (*Alumen*); Amm-c. (*Ammonium Carbonicum*), with Ammonc. (*Ammoniacum*). Lastly, in vol. V, p 588, we find a proving of *Linum Catharticum*, or Purging Flax, under the name of *Linum;* and in vol. X, p. 574, a proving of *Linum Usitatissimum* (Linseed), under the same name, *Linum;* and the symptoms of the two are all jumbled up together in the *Index*.

I have found other errors in these works which I have sent to Dr. Lee to use in the compilation of his *Repertory—the very best the world has ever seen.*

OTHER ERRATA.

*Lippe's Repertory*, p. 220, > by warmth of bed, for Am-c. read Am-mur.; p. 170, croup. ? transpose after and before midnight; see Kent in M. A., p. 309.

HOMŒOPATHIC PHYSICIAN, vol. IX, p. 295, line 8. ? for tumor read tremor.

*Allen's Index to Encyclopædia*, p. 1175, burning in thumb, for Arum read Arundo-m.

